# Unraveling the pathogenesis of Barrett’s esophagus and esophageal adenocarcinoma: the “omics” era

**DOI:** 10.3389/fonc.2024.1458138

**Published:** 2025-01-30

**Authors:** Alberto Barchi, Giuseppe Dell’Anna, Luca Massimino, Francesco Vito Mandarino, Edoardo Vespa, Edi Viale, Sandro Passaretti, Vito Annese, Alberto Malesci, Silvio Danese, Federica Ungaro

**Affiliations:** ^1^ Gastroenterology and Digestive Endoscopy, IRCCS Ospedale San Raffaele, Milan, Italy; ^2^ Gastroenterology and Gastrointestinal Endoscopy Unit, IRCCS Policlinico San Donato, Milan, Italy; ^3^ Università Vita-Salute San Raffaele, Faculty of Medicine, Milan, Italy

**Keywords:** Barrett esophagus, esophageal cancer, metaplasia, omics, genomics, transcriptomics, epigenomics, miRNAs

## Abstract

Barrett’s esophagus (BE) represents a pre-cancerous condition that is characterized by the metaplastic conversion of the squamous esophageal epithelium to a columnar intestinal-like phenotype. BE is the consequence of chronic reflux disease and has a potential progression burden to esophageal adenocarcinoma (EAC). The pathogenesis of BE and EAC has been extensively studied but not completely understood, and it is based on two main hypotheses: “transdifferentiation” and “transcommitment”. Omics technologies, thanks to the potentiality of managing huge amounts of genetic and epigenetic data, sequencing the whole genome, have revolutionized the understanding of BE carcinogenesis, paving the way for biomarker development helpful in early diagnosis and risk progression assessment. Genomics and transcriptomics studies, implemented with the most advanced bioinformatics technologies, have brought to light many new risk loci and genomic alterations connected to BE and its progression to EAC, further exploring the complex pathogenesis of the disease. Early mutations of the TP53 gene, together with late aberrations of other oncosuppressor genes (SMAD4 or CKND2A), represent a genetic driving force behind BE. Genomic instability, nonetheless, is the central core of the disease. The implementation of transcriptomic and proteomic analysis, even at the single-cell level, has widened the horizons, complementing the genomic alterations with their transcriptional and translational bond. Increasing interest has been gathered around small circulating genetic traces (circulating-free DNA and micro-RNAs) with a potential role as blood biomarkers. Epigenetic alterations (such as hyper or hypo-methylation) play a meaningful role in esophageal carcinogenesis as well as the study of the tumor micro-environment, which has led to the development of novel immunological therapeutic options. Finally, the esophageal microbiome could be the protagonist to be investigated, deepening our understanding of the subtle association between the host microbiota and tumor development.

## 1 Introduction

### 1.1 Barrett’s esophagus as a complication of reflux disease: epidemiology and risk factors

Barrett’s esophagus (BE) is a pre-cancerous condition characterized by the transition of the squamous mucosa of the esophagus to a metaplastic columnar intestinal epithelium as a defensive mechanism against the chronic insult of gastric acid reflux (1). Upper GI endoscopy is still the most important tool for the detection of BE, and the diagnosis needs to be ascertained by a pathologist after a comprehensive biopsy protocol execution (2). BE incidence is slowly increasing in developed countries, with a current estimate of around 2% per patient-year (3). BE is mostly associated with gastro-esophageal reflux disease (GERD), affecting 10% of all chronic reflux patients worldwide (4). If BE incidence is low, esophageal adenocarcinoma (EAC) incidence is lower (0.5% per patient-year), even though this percentage could be hindered by the inclusion of ultra-short (<1 cm) Barrett or gastric-cardia-intestinal metaplasia (IM) (5). Several risk factors are implied in the development of BE and its progression to EAC. BE is widely accepted as a GERD complication; therefore, GERD-related features such as typical symptoms and their length (6), acid/bile reflux burden (7, 8), large hiatal hernias (9), and hypotensive lower esophageal sphincter (LES) (10) present a strong correlation with BE development. Meanwhile, if *Helicobacter pylori* (Hp) is believed to be a protective factor for BE (11) due to the decreased gastric acid output following gastric chronic inflammation, male sex (12), smoking habit (13), and obesity (14) account for an increased risk of BE, while GERD treatment and vegetable consumption lower its burden (15).

### 1.2 Diagnosis, staging, and management

Endoscopy with histological confirmation of IM is still the gold standard for BE diagnosis (16). The most recent European guidelines confirmed the importance of endoscopic screening in a selected population of patients >50 years with chronic reflux symptoms despite proton pump inhibitor (PPI) therapy. Moreover, if a family history of BE or EAC is present (17), mono or double therapy with PPIs is always advised as chemoprevention in patients with confirmed BE. BE diagnosis begins with a proper endoscopic evaluation, clearly stating the length and circular extension of the columnar metaplastic epithelium following the Prague classification criteria (18). A minimum circular extension of 1 cm or a length of 2 cm is necessary to define the presence of BE and guide the subsequent management (17). A comprehensive endoscopic assessment of columnar and squamous esophageal epithelium must imply firstly white light (WL) evaluation implemented with the most advanced post-processing imaging techniques, primarily narrow band imaging (NBI) (19). The accuracy of NBI in detecting early dysplastic lesions within the BE mucosa is known since several years, and it has been improved by recent advancements in endoscopic equipment (20). After a thorough examination, four-quadrant biopsies should be collected at every 2 cm of BE mucosa to lead to a comprehensive pathology staging in spotting untargeted dysplastic areas (17). The surveillance after BE diagnosis is mainly pivoted by length and circular extension (advised every 5 years with <3 cm in length) and by random dysplasia detection (suggested within 6 months after confirmed low-grade dysplasia [LGD] and 3 months after a high grade [HGD] diagnosis (17). Every visible lesion at endoscopy examination suspected of dysplasia should be resected and the remnant BE ablated after a thorough disease staging via positron emission tomography–computed tomography (PET-CT) and endoscopic ultrasound (EUS) if needed. Endoscopic resection and subsequent careful surveillance are indicated for every EAC confined to the submucosal layer without lympho-vascular invasion and no poor tumor differentiation (17).

## 2 Pathogenesis

**Figure 1:**
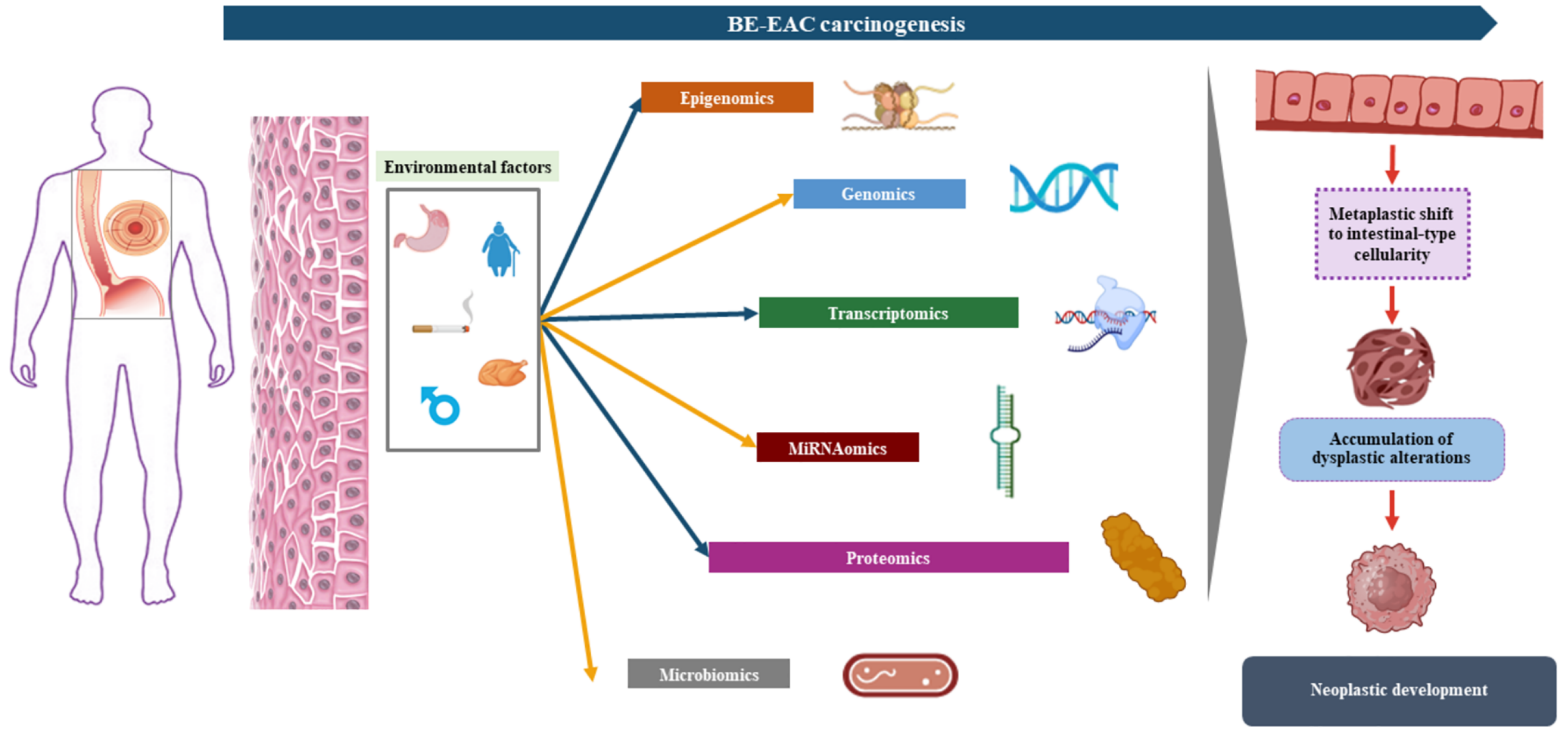
Barrett’s esophagus (BE) pathogenesis in relation to acid bile reflux initial trigger, with the display of the two main development hypotheses: “trans-commitment” of stem cells from submucosal glands or the basal esophageal epithelium via upregulation of sonic hedgehog signaling followed by SMAD/BMP4 over-expression, contributing to the production of cells presenting mucins and cytokeratins related to columnar intestinal epithelium (MUC2/CDX2); “trans-differentiation” moving from the reflux insult causing the production of high levels of reactive oxygen species which cause DNA damage (mostly double-strand breaks) and the induction of a pro-inflammatory environment, promoting the downregulation of NOTCH signal and ultimately leading to squamous–columnar metaplastic cell differentiation.

Despite optimization of clinical assessment and technological advancements, BE diagnosis still represents a sword of Damocles for patients who have to face multiple treatments and frequent endoscopic surveillance. Therefore, understanding of BE pathogenesis with its subtle molecular mechanisms of development and progression is pivotal (21). Metaplastic changes in BE are primarily driven by the chronic acid/bile insult on the squamous epithelium, inducing genetic and epigenetic modifications in the cell lineage (22). BE mucosa resembles the small intestine architecture with crypts displaying several mucus-secreting cells, expressing MUC1, MUC2, MUC3, or MUC5, either goblet-like or columnar-shaped, together with the trefoil family member cells TFF1, TFF2, and TFF3 (22). The two main hypotheses on metaplastic changes, reflux-induced mechanisms, are the “transdifferentiation” and the “transcommitment” models (**Figure 1**). Concerning the first model, IM could develop following the pro-inflammatory environment created by chronic reflux injury. The main principle by which reflux induces metaplastic conversion of the esophageal mucosa is oxidative stress, with the over-production of different toxic products, primarily reactive oxygen species (ROS) (1). The inflammatory environment deriving from the damage mediated by these products may lead to an increase in oxidative DNA damage (and related markers such as p-H2AX) (22) via TNFα, prostaglandin-E2 (PGE2), and the nuclear factor-κB (NF-κB) production. Interestingly, the damages caused by oxidative stress, mainly double-strand DNA breaks, lead to the upregulation of several enzymes, most importantly poly(ADP-ribose) polymerase (PARP)-1, with purposes of DNA repair (22). PARP-1 exploits its duties via NAD^+^ production, which ultimately consumes ATP, leading to cell death and re-programming. All of these processes could lead to a differentiation of the squamous epithelium to a firstly non-specialized columnar epithelium containing glands showing the overexpression of several phosphorylated SMAD proteins via sonic hedgehog signaling upregulation (23). This initial differentiation has been reported in mice overexpressing Bmp4 (from the TGF beta family) together with SMAD proteins (23). This pathway eventually leads to the overexpression of the intestinal marker CDX2, a key component of IM cell lineage, indicating a more specialized columnar epithelium. CDX2 overexpression has been detected in biopsy samples from reflux patients with IM compared to non-IM patients (24). The different cell types present in the BE mucosa are more deeply explained by the “transcommitment” hypothesis. Submucosal esophageal glands harbor pluripotent stem cells which may undergo reprogramming for columnar-type differentiation via a Notch1-dependent mechanism (25). This mechanism has been recently unraveled via single-cell RNA sequencing (scRNA-seq) in BE patients compared to healthy volunteers (HV) (26). Another plausible mechanism involves the re-programming of residual embryonic stem cells from the squamo-columnar junction (SCJ), characterized by the expression of KRT7. The development of BE-related IM from the SCJ stem cell niche has been described in a CDX2-overexpressing transgenic mouse model (27). BE is a complex mechanistic transformation and possibly involves all of these described mechanisms.

## 3 The “omics” revolution in risk progression assessment and biomarker development

**Figure 2:**
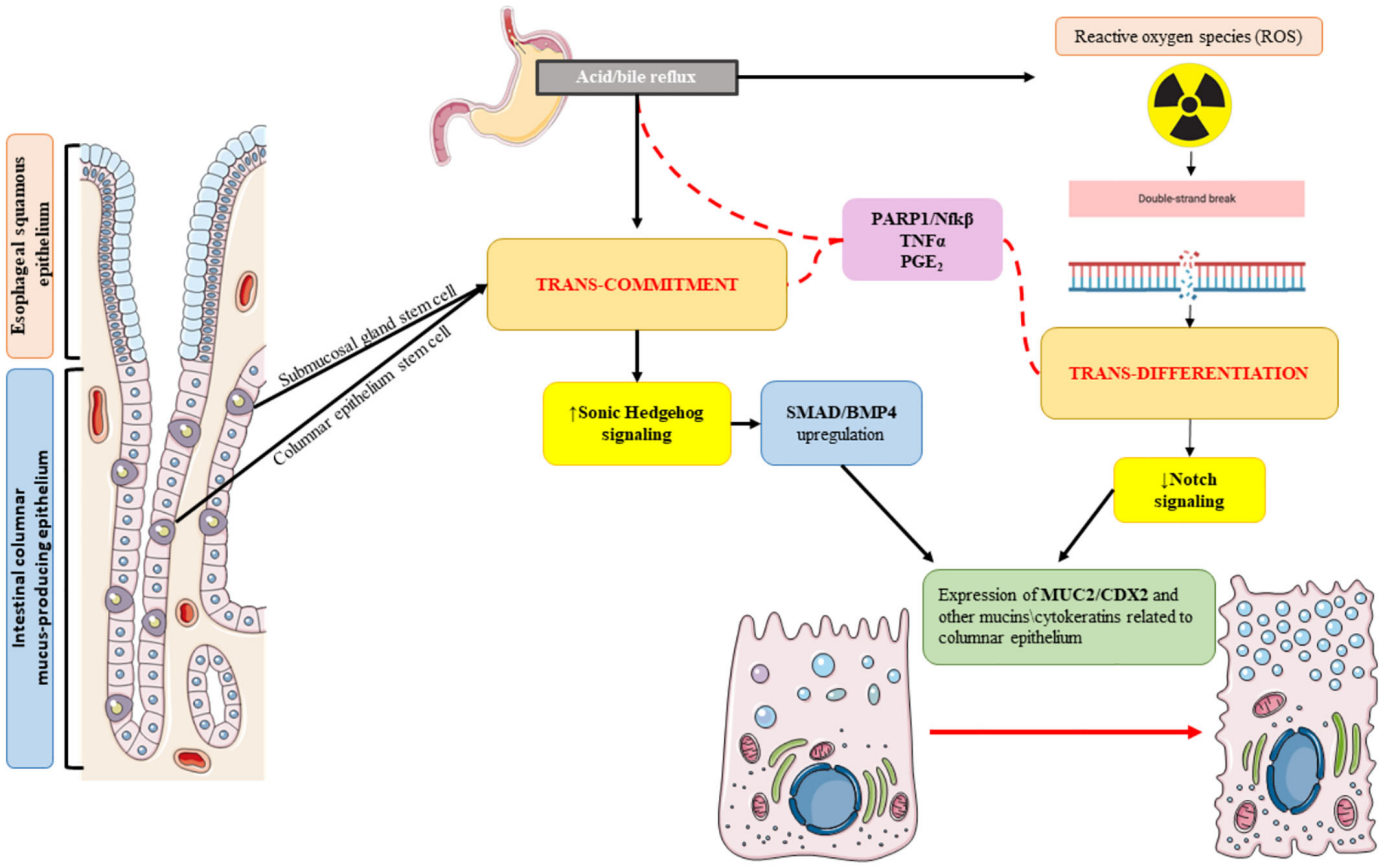
Barrett’s esophagus (BE) carcinogenesis process addressed thanks to “omics” approach: the integration of the various omics fields (genomics, transcriptomics, proteomics, miRNAomics, and microbiomics) is crucial in unveiling the subtle molecular mechanisms leading to the metaplastic shift of the squamous esophageal epithelium to columnar intestine-like BE epithelium, the occurrence of dysplasia, and the development of invasive cancer.

Since 1990, the year of the first sequencing of the whole human genome, new high-throughput sequencing technologies have been developed to address the different “omics” fields: the analysis of DNA (genomics), transcribed RNA (transcriptomics), proteins expression (proteomics) as well as epigenomics and microbiomics characterization (28). Carcinogenesis profiling by means of each of these technologies has provided further depth to BE and progression to EAC, but their combination has been even more effective (**Figure 2**). The “multi-omics” approach is central in nowadays’ basic research to explore the risk profiles of BE and EAC and to identify new potential biomarkers helpful in early non-invasive diagnosis and tumor treatment response assessment.

### 3.1 Genomics- and transcriptomics-based studies

The importance of early detection is central in the management of BE. The cumulative risk of BE progressing to EAC ranges from 0.1% to 0.35% (29). Therefore, unraveling the progression of BE to dysplasia is pivotal. Genomics has played a central role in studying potential pathways of BE progression. Genome-wide technologies have made an outbreak in basic and translational research as powerful instruments to characterize the genotype–phenotype association for various diseases or traits (30) (**Table 1**). Genome-wide association studies (GWAS) are studies where alleles of particular genes containing micro-variations in their genetic code (the so-called single-nucleotide polymorphisms, SNPs) are associated with specific phenotypic features of a known polygenic multifactorial disease, such as BE and EAC (30). Genotyping in GWAS can be performed with SNP arrays or via whole-genome sequencing (WGS) (30). GWAS take the first step from the collection of extensive quantities of genomic data on patients in large cohort studies (30). The genotyping of SNPs is usually done with the two most common arrays (Affymetrix and Illumina platforms), with the latter being the most commonly used. Functional characterization through complex computational analysis serves the purpose of identifying clear causal relations of the evidenced SNPs with clinical traits. A rigid technical and genetic quality control and SNP imputation are performed before the actual analysis in order to ensure the quality of the phenotype associations for a very high number of polymorphisms at the same time (30). The pooled analysis through meta-analytic approach of data comprised in multiple GWAS has further strengthened their scientific soundness and reliability. Ek W.E. and colleagues in 2013 evaluated “unrelated” heritability (*h* ^2g^), defined as the amount of phenotypical variation explained by the co-integration of the most associated SNPs of either BE and EAC (35% and 25% respectively, *p <*1 × 10^9^ and *p <*1 × 10^7^). The authors identified also a significant genetic correlation (r_g_) at bivariate analysis between BE and EAC firstly pointing out a likely shared germline susceptibility (32). Moreover, a consistent polygenic overlap was demonstrated between BE and EAC, but not significant with GERD (32). These data on unrelated individuals firstly hinted at the underlying shared genetic pattern of BE and EAC, differently from GERD, deepening in some ways the results of the just previously published first GWAS on BE (31). Su et al. have found two SNPs, one in the major histocompatibility complex (MHC) locus (*rs9257809*; odds ratio [OR] = 1.21) and one close to the FOXF1 gene (*rs9936833;* OR = 1.14) as strongly correlated with BE (31). Aside from FOXF1, FOXP1 has also been unraveled as a potential gene correlated with esophageal development regulation and with BE and EAC development (33). Dai et al. explored the association between several environmental risk factors for BE progression with the SNP *rs2687201* in the FOXP1 gene, finding a consistently increased correlation of reflux symptoms with BE in patients homozygous for the major allele of *rs2687201* (no significance for BMI and smoking status was detected) (33). Another relevant study by Buas et al. explored and confirmed a strong association with BE for several SNPs implied in the cyclooxygenase (COX) and oxidative stress pathway (specifically the MGST1 gene), further strengthening the power of the correlation (*p* < 5.5 × 105) through a meta-analysis (36). This study evaluated the same cohort already exploited by Levine and colleagues who identified CRTC1, BARX1, and FOXP1 as related with BE (21). The largest-scale meta-analytic effort to date encompassed all the largest SNP-array GWAS on BE and EAC, identifying eight novel risk loci (among which are CFTR, MSRA, LINC00208, KHDRBS2, TPPP, CEP72, TMOD1, and SATB2), and firstly evaluated a highly correlated disease pathway comprising muscle lineage differentiation and epithelial–mesenchymal transition through functional characterization (35). Recently, an implementation of this study unraveled 11 additional risk loci and, through polygenic risk scoring (PRS), delineated promising risk models for BE development (40). Notably, the authors also provided a meta-analysis of transcriptomic signatures, reporting five more risk loci reaching transcriptome-wide significance (40). Genomics studies also helped in unveiling the risk of progression of BE to EAC, fostering novel trajectories to foster novel biomarkers (43). The exploration of genome-based technology has also expanded forward the role of SNPs. Through whole-genome sequencing (WGS), it was assessed that EAC occurrence is most likely related to early mutations of TP53 gene, even in non-dysplastic BE, followed by genomic duplication driving the late stages of the cancer cascade, as confirmed by exome sequencing (34). Furthermore, the high frequency of somatic mutation even in non-dysplastic BE was strengthened as previously reported by relevant genomics studies (up to 1.3–5.4 mutations per Mb) (44, 45). These results somewhat confirmed that only a few cases of BE–EAC oncogenic development are actually based on the usual oncologic suppressor genes (TP53, SMAD4, or CDKN2A) followed by oncogene amplification, while it seems more likely that genetic diversity is maintained throughout the process (46). Chromosomal alterations have been described as potential markers of BE progression (43). Aneuploidy, the abnormal number of cell chromosomes, has been identified as the only predictor of histologic progression in a cohort of 127 BE patients, with a 6.6-fold risk increase (38). The accuracy of aneuploidy was low, probably due to technique issues (flow cytometry on freshly frozen samples against the formalin-fixed paraffin-embedded biopsies [FFPE] standard-of-care techniques). Copy number alterations (CNAs), markers of genomic instability, have equally been studied in BE and EAC (43). Genomic disorder, characterized by an accumulation of CNAs, has been shown to be strongly related to BE progression, and a recently built model on the degree of instability and CAN number reported good overall accuracy (area under the curve [AUC] of 89%) (39). More recently, Bao and colleagues, using WGS, identified initial TP53 inactivation as the first step leading to increasing genomic instability in BE progression (41). In the same study, the authors also found whole-genome duplication (WGD) as a significant trigger of the rapid accumulation of CNAs, leading to cancer (41). Even chromosomal translocations have been described in the progression of BE. A recurring translocation (t10:16) between chromosomes 2, 10, and 16 has been found in BE cells just before their malignant conversion (47). These genomic aberrances are frequently detected in selected locations called tracts of homozygosity (TOH), homozygous genotyped stretches of DNA already associated with insertion, deletion, SNPs, and CNAs in humans (48). A recent study by Wanchai et al., through a genome-wide analysis, has identified 24 TOH regions (13 over-represented and 11 under-represented) and described the occurrence of genomic alterations within them, particularly insertion/deletions, inversed repeats (IRs), and SNPs (37). At gene mapping, 33 genes were found to be correlated with genomic abnormalities, among which WDR63 carried the most weight (37). Moreover, 28 among 33 were proved to be involved in tumorigenesis pathways and cancer progression through network analysis (37). Single-cell sequencing (SCS) has burst in recent years, revolutionizing the understanding of genotype–phenotype bonds (49). Applying “omics” analysis at the single-cell level could unveil how genomic alterations can affect transcriptomic expression in various diseases, including cancer (50). Sc-RNA sequencing analyses have previously reported a transcriptional overlap between BE and gastric cells (antral and pyloric) (51) and identified genes (particularly TFF3, SPINK4, and others) specific for BE (26). A recent comprehensive analysis aiming at the integration of sc-RNA and sc-DNA sequencing data from BE tissues, through a new bioinformatics model (the “MaCroDNA”), enhanced the intratumor heterogeneity of HER2 expression via ERBB2 gene mutations (as CNAs) (42). The integration of single-cell genomics and transcriptomics data could be the future of oncologic research in BE/EAC. Another relevant field in BE and EAC prevention is blood-cell-free DNA (cf-DNA) assessment. The cancer association of cf-DNA dated back in 1977 (52), and from that time its knowledge has considerably widened. The big issue of blood cf-DNA is that in BE or in the early stages of EAC the abundance of cf-DNA is relatively low (53). A metanalysis showed that cf-DNA assays provided low sensitivity both in the diagnosis (71%) as in prognosis and monitoring (48.9%) (54). Among the 15 studies included, the majority focused on next-generation sequencing (NGS) as the preferential technique for cf-DNA assessment (53). Even though cf-DNA-based studies, particularly for treatment response and EAC prevention, showed promising results (55, 56), further improvements in test accuracy and sensitivity must be made.

**Table 1:** Genomics and transcriptomics studies in BE and EAC.

Study	Omics domain	Platform	Sample (cases/controls)	Outcomes	Target genes
Su Z. et al., 2013 (31)	Genomics	SNP array	1,852/5,172 “discovery” cohort 5,986/12,825 “replication” cohort	*rs9257809* [*p* = 4.09 × 10^−9^, OR = 1.21 (1.13–1.28) at 6p21]	MHC
				*rs9936833* [*p* = 2.74 × 10^−10^, OR = 1.14 (1.10–1.19) at 16q24]	FOXF1
Ek W.E. et al., 2013 (32)	Genomics	SNP array	1,509 (EA) + 2,383 (BE)/2,170 1,445 (GERD)/2,349 for the polygenic risk cohort	BE: h^2g^ 35%; SE = 6%; *p* = 1 × 10^−9^ EA: h^2g^ = 25%; SE = 5%; *p* = 2 × 10^−7^ R_g_(BE-EA) = 1.0; SE = 0.37 Polygenic overlap BE-EAC: *p* = 1 × 10^−6^	–
Levine D.M. et al., 2013 (21)	Genomics	SNP array	3,175 (BE) + 2,390 (EAC)/10,120	SNP *rs10419226* at 19p13 (*p* = 3.6 × 10^-10^); *rs11789015* at 9q22 (*p* = 1.0 × 10^-9^) and r*s2687201* at 3p14 (*p* = 5.5 × 10^-9^)	CRTC1, BARX1, FOXP1
Dai et al., 2015 (33)	Genomics	SNP array	1,516 (BE) + 2,416 (EAC)/2,187	Minor alleles of *rs2687201* increased the BE risk in the presence of GERD symptoms (*p* < 0.0005) Major allele homozygosity strongly associated with BE compared to minor alleles (OR 6.17 vs. OR 5.44)	FOXP1
Stachler D.M. et al., 2015 (34)	Genomics	WGS	25 BE/EAC patients	Genomic disruption is the driver of BE progression. Increasing number of deletions (*p* < 0.01) and amplifications (*p* < 0.001) from BE to EAC Homozygous CDKN2A deletion (with p53 loss) in 4/11 BE TP53 mutations determined in early cancers followed by genomic doubling	CDKN2A, TP53
Gharahkhani P. et al., 2016 (35)	Genomics	SNP array	6,167 (BE) + 4,112 (EAC)/17,159	Metanalysis of all GWAS cohorts published until 2016 8 new SNPs identified: *rs1745175* (*p* = 4·8 × 10^-10^), *rs17749155* (*p* = 5·2 × 10^-10^), *rs10108511* (*p* = 2·1 × 10^-9^), *rs62423175* (*p* = 3·0 × 10^-9^), *rs9918259* (*p* = 3·2 × 10^-9^), *rs7852462* (*p* = 1·5 × 10^-8^), *rs139606545* (*p* = 2·0 × 10^-8^), *rs9823696* (*p* = 1·6 × 10^-8^) Most significant pathway for BE (*p* < 10^-6^): muscle and mesenchyme differentiation	CFTR, MSRA, LINC00208, BLK KHDRBS2, TPPP, CEP72, TMOD1, SATB2, HTR3C and ABCC5
Buas et al., 2017 (36)	Genomics	SNP array	3,295 (BE) + 2,515 (EAC)/3,207	Significant association of COX pathway and BE (*p* = 0.0059)	MGST1
Wanchai et al., 2019 (37)	Genomics	WES	176/192	24 TOH regions: 13 over-represented and 11 underrepresented compared to controls High abundance of IRs in chromosome 2 and 9 TOH regions Chromosome 7 and 15 TOH regions showed the longest insertions/deletions At gene mapping, 33 genes related to genomic alterations (of which seven highly correlated with cancer genesis)	WD63, VPS13B, MON2, CTAGE5, GNA12, KAT2B, RBMS3, TLE1
Hadjinicolaou A.V. et al., 2020 (38)	Genomics	IHC, Microsatellite array, PCR, flow cytometry	127 (BE) (42 progressors, 85 non-progressors)	Multi-marker molecular assay on long-term follow-up BE cohort Aneuploidy (*p <* 0.0008) and p53 (*p <* 0.038) were significant predictors of progression	p53
Killcoyne S. et al., 2020 (39)	Genomics	WGS	88 (BE)	CNAs patterns associated with BE not progressed and progressed to dysplasia were used to build risk model AUC of 89% in predicting progression	–
Schroder J. et al., 2023 (40)	Genomics Transcriptomics	SNP array	11,208 (BE) + 5,582 (EAC)/32,476	GWAS: high genetic correlation between BE and EAC (R_g_ = 0.92, *p* =1.8×10^−75^), *h* ^2g^ of BE and EAC (21.7% and 15.0%, respectively); 24 significant SNPs of which 11 were novel TWAS: five novel loci not identified by the GWAS Significant genetic correlation (R_g)_ of GERD, obesity, HH and educational attainment with BE/EAC PRS: The combined risk model—including PRS, PCs, genotyping array, age, sex, BMI, hiatal hernia and GERD—achieved an AUC of 80.20 ± 0.005 for BE/EA, 80.61 ± 0.005 for BE and 77.09 ± 0.015 for EA	FMNL2, SLC66A1L, SPRY1, NNT, ARL15, TFAP2A, SLC25A21, NR2F2, JAG1
Bao C. et al., 2023 (41)	Genomics	WGS	15 (BE, dysplastic, EAC)	Bi-allelic TP53 inactivation is an early event Focal deletion near FHIT gene Bi-allelic deletion of CDKN2A Higher number of CNAs are related to genome duplication, driving to multi-generational aneuploidy and chromosomal instability	TP53, FHIT, CDKN2A
Edrisi M. et al., 2023 (42)	Genomics Transcriptomics	Sc-DNA seq Sc-RNA seq	–	Pearson correlation for quantifying the association between sc-RNA seq cells with sc-DNA seq cells, using MaCroDNA assay MaCroDNA identified a phylogenetic signal of the HER2-encoding gene *ERBB2* in dysplastic and non-dysplastic BE	ERBB2

AUC, area under the curve; BE, Barrett’s esophagus; BMI, body mass index; CNA, copy number alteration; COX, cyclo-oxygenase; EAC, esophageal adenocarcinoma; GERD, gastroesophageal reflux disease; GWAS, genome-wide association study; HH, hiatal hernia; H^2g^, unrelated genetic heritability; IR, inverted repeats; OR, odds ratio; PC, principal component; PCR, polymerase chain reaction; PRS, polygenic risk scoring; Sc D(R)NA seq, single-sell D(R)NA sequencing; SE, standard error; SNP, single-nucleotide polymorphism; TOH, tract of homozygosity; TWAS, transcriptome-wide association study; WGS, whole-genome sequencing; WES, whole-exome sequencing.

### 3.2 Micro-RNA profiling

Micro-RNAs (miRNAs) are a class of small non-coding RNAs which have been investigated as cancer biomarkers in several settings (57). miRNAs are usually ~20 bp in length and able to target several coding and non-coding peripheral regions in the DNA, controlling gene regulation and protein expression (58). Usually, mi-RNAs are assessed in the serum of patients through the use of RT-PCR with dedicated assays (as TaqMan Micro-RNA kit). First, mi-RNA assays assessed the different expression abundances of miRNAs in BE and EAC compared to controls (59). miR-200, miR-141, and miR196 were among the first miRNAs directly associated with alterations in cell proliferation, apoptosis, and cell migration processes (60, 61). miR-192 and miR-215 have been detected to be overexpressed in BE progressing to EAC, being targeted by p53 pathway, leading to cell cycle arrest (62). Furthermore, the overexpression of miR-21 has been shown to embody an oncogenic role via the negative regulation of PTEN, TMP1, and other pathways (63). Concerning mi-RNAs exploiting an onco-suppressant role, the Let-7 family has been widely described as downregulated in BE progressing to EAC (64). Along with the recent outburst of “omics”, further attention has been focused on genetic abnormalities within different mi-RNAs. An Italian group recently evaluated SNPs near mi-RNA biogenetic genes or mi-RNA gene loci through Illumina sequencing on previously published BE/EAC datasets (65), reporting 29 and 25 SNPs to be associated with EAC and BE risk, respectively (66). Interestingly, the authors also explored SNPs related to mi-RNA–mRNA interaction, unraveling the relevance of mi-RNAs in transcriptional regulation processes (66). With the same methodological design, Yao et al. identified 21 novel differentially expressed mi-RNAs affecting mRNA transcription in BE progressing to EAC, further analyzing the biological processes targeted by these mi-RNAs through Gene Ontology (GO) analysis (67). Further efforts must be made for the implementation of miRNA-omics in the screening and monitoring of BE/EAC.

### 3.3 Epigenomics in BE profiling

Epigenetic modifications, encompassing different DNA manipulations (methylation, hydroxylation, formylation, and carboxylation), chromatin marking, and histone modification, regulate the transcription of various genomic regions controlling cellular activity (68). Growing evidence highlights the role of epigenetic alterations in gastrointestinal (GI) tract tumors and precancerous lesions, including BE and EAC (69). In this context, DNA methylations have been the most extensively studied (70). DNA hyper-methylation promotes cancer progression by downregulating oncosuppressor genes. In BE and EAC, hyper-methylated genes include several oncosuppressors, particularly APC, CDKN2A (p16INK4a), RUNX3, MGMT, CDH1, and SFRP (71, 72). In 2019, Yu M et al. published the results of a genome-wide methylation analysis, identifying four subtypes of BE and EAC based on methylation grade: high, intermediate, low, and minimal (73). Jammula S et al. published a retrospective cohort study, including tissue samples and clinical data from 150 BE and 285 EAC cases within the Oesophageal Cancer Classification and Molecular Stratification Consortium (OCCMSC) in the UK (74). The authors identified four subtypes of BE and EAC based on DNA methylation profiles, integrating transcriptomics and genomics data. Subtype 1 was characterized by DNA hyper-methylation, high mutational burden, and multiple alterations in cell cycle and receptor tyrosine signaling pathway genes (CDKN2A, GATA4, CCND1, and ERBB2 CNAs). Subtype 2 was associated with metabolic processes (ATP synthesis and fatty acid oxidation) and lacked methylation at specific transcription factor binding sites (in the CDKN2A gene); 83% of this subtype were BE and 17% were EAC. Subtype 3 showed no changes in methylation compared to control tissue but had a gene expression pattern indicating immune cell infiltration associated with the shortest patient survival. Finally, subtype 4 presented DNA hypo-methylation linked with structural rearrangements and CNAs, with preferential amplification of CCNE1; these cells were sensitive to CDK2 inhibitors (74). Growing evidence concerns the identification of tumor biomarkers in EAC, addressing the lack of early diagnostic markers and prognostic factors. Identifying genes and loci targeted by DNA methylation has promoted them as potential screening and surveillance biomarkers in this setting (**Table 2**).

**Table 2:** DNA methylation biomarkers for Barrett’s esophagus and esophageal adenocarcinoma.

Reference	Biomarker	Study design	Samples (case/control)	Results	Clinical application
Moinova H.R. et al., 2018 (75)	CCNA1, VIM	Observational study	173 + 149 (validation cohort)	CCNA1 with AUC 0.95 (BE vs. controls) CCNA1 + VIM showed 90.3% sensitivity and 91.7% specificity	BE screening
Yu M. et al., 2015 (73)	B3GAT2, ZNF793	Observational study	98 + 66 (validation cohort)	B3GAT2 - ZNF793 methylation rates: 32.5% - 31.1 (BE patients) vs. 2.19% - 2.52 (controls) (*p* < 0.0001)	BE screening
Chettouh H. et al., 2018 (76)	TFPI2	Pilot study	30 + 278 (validation cohort)	TFPI2 sensitivity 82.2%–specificity 95.7%	BE screening
Maity A.K. et al., 2022 (77)	CTNND2, CCL20	Retrospective study	407	CTNND2 inactivated in BE – CCL20 overactivated in EAC	EAC screening
Schulmann K et al., 2005 (78)	p16, RUNX3, HPPI	Retrospective study	234	p16 (OR 1.74, 95%CI 1.33–2.20), RUNX3 (OR 1.80, 95%CI 1.08–2.81), HPPI (OR 1.77, 95%CI 1.06–2.81)	EAC screening
Jin Z. et al., 2009 (79)	p16 + RUNX3 + HPP1 + NELL1 + TAC1 + SST + AKAP12 + CDH13	Retrospective study	195	Sensitivity 50%–specificity 90%	Progression risk
Alvi M.A. et al., 2013 (80)	SLC22A18, PIGR, GJA12, RIN2	Retrospective study + prospective multicentric study	186 (retrospective cohort), 135 (prospective cohort)	AUC 0.98	EAC screening

BE, Barrett’s esophagus; EAC, esophageal adenocarcinoma; AUC, area under the curve; OR, odds ratio; CI, confidential interval.

#### 3.3.1 Epigenetic biomarkers for BE and EAC screening

Since coming up with new potential biomarkers for early diagnosis is crucial in EAC, epigenomics have shown promising results (43). In 2018, Moinova HR et al. published results from a study on a biomarker-based, non-endoscopic method to detect BE (75). This method utilized a swallowable balloon-based esophageal sampling device that detects methylated DNAs. Using this device, the authors performed cytology brushing of the distal esophagus in 173 patients (with and without BE) to test CCNA1 DNA methylation as a potential BE biomarker. Compared to non-affected patients, CCNA1 DNA methylation showed an area under the curve value of 0.95 for distinguishing BE-related metaplasia and neoplasia cases. When the results of the two biomarkers were combined, the sensitivity was 95% and the specificity was 91% (75). In 2015, Yu M et al. conducted a genome-wide methylation analysis to identify potential BE biomarkers in endoscopic brushing samples from squamous epithelium, stomach, and BE (81). They compared the methylation levels at over 485,000 CpG sites using pyrosequencing and MethyLight assays. Their findings revealed that the B3GAT2 and ZNF793 methylation rates were significantly higher in BE samples (32.5% and 33.1%, respectively) compared to control samples (2.29% and 2.52%, respectively) (*p* < 0.0001) (81). The Esocheck device used in the above-mentioned studies allowed sampling only from the distal esophagus. Therefore, other swallowable cytology collection devices, such as Cytosponge and Esochacap, were introduced to sample the entire esophagus and oropharynx (82). Cytosponge was evaluated in a recent study involving a pilot cohort (20 BE cases and 10 controls) and a validation cohort (149 BE cases and 129 controls) from the Cytosponge BEST2 trial (ISRCTN12730505) (76, 82). The hyper-methylation rates of TFPI, TWIST1, ZNF354, and ZNF569 were significantly higher in the BE samples than in the controls (*p* < 0.001), and these findings were confirmed on histology from the same cohorts (76). Maity AK et al. used systems epigenomic and cell type deconvolution methods on RNA-Seq and DNA methylation data from EAC patients and their adjacent normal tissues to identify reliable biomarkers unaffected by cell type heterogeneity (77). The authors discovered 12 gene modules epigenetically deregulated in EAC and validated these findings in four independent EAC cohorts. Single-cell RNA-Seq data revealed that one module, centered around CTNND2, was inactivated in BE. Additionally, DNA methylation patterns in saliva from EAC patients identified a chemokine module centered around CCL20, correlating with EAC status. According to these findings, CTNND2 and CCL20 modules resulted in promising biomarkers for EAC and warrant further research (77).

#### 3.3.2 Epigenetic biomarkers for BE follow-up and risk stratification

Currently, there are no biomarkers available in clinical practice to stratify BE patients and predict their risk of progression, except for p53 and aneuploidy, which could potentially reduce the overall burden of endoscopic follow-up (83). Schulmann et al. investigated the role of epigenetic modifications in terms of risk progression, comparing BE patients who developed cancer with ones who did not (78). Hypermethylation of p16 (OR 1.74; 95%CI, 1.33–2.20), RUNX3 (OR 1.80; 95%CI, 1.08–2.81), and HPPI (OR 1.77; 95%CI, 1.06–2.81) was associated with an increased progression risk (78). In a subsequent study, Sato F et al. developed a multi-parameter stratification tool that combined clinical factors (sex, BE length, and pathological evaluation) with previously identified epigenetic biomarkers (CDKN2A, RUNX3, and HPPI) (84). This tool identified three distinct BE patient groups: high, intermediate, and low risk of progression (84). More robust data come from a retrospective, double-blinded validation study that included 195 BE patients (both cancer developers and non-developers) (79). Eight methylation biomarkers (p16, RUNX3, HPP1, NELL1, TAC1, SST, AKAP12, and CDH13) were used to create a progression risk panel with sensitivity of 50% and specificity of 90% (79). A four-gene methylation panel was proposed by Alvi MA et al. in a prospective multicenter study (80). The authors began by identifying 27K methylation arrays to find genes differentiating between BE and EAC samples. This was validated by pyrosequencing firstly on a retrospective cohort (60 BE, 36 LGD/HGD, and 90 EAC) and then on a prospective one (60 BE, including 28 LGD/HGD and nine EAC). Four genes (SLC22A18, PIGR, GJA12, and RIN2) showed an AUC of 0.98 for differentiating between BE and EAC. This panel was able to stratify prospective cohort patients into three risk groups based on the number of methylated genes (low risk: <2 genes, intermediate: 2, and high: >2) (80).

### 3.4 TME composition

The tumor microenvironment (TME) is primarily composed of two distinct subsets of cells: immune cells (such as T cells, B cells, and macrophages) and mesenchymal cells (overall cancer-associated fibroblasts or CAFs). The extracellular matrix (ECM) regulates the interaction between the TME and the immune system through CAFs (85). The CAFs, recruited to the tumor site by TGF-beta and CXCR4/CXCL12 signaling, enhance cancer cell proliferation and polarize adaptive and innate immune cells toward a tumor-promoting phenotype (also conferring immunotherapy resistance) (86).

T cells represent another crucial component of TME. CD4+ T helper cells are classified into different subsets based on their specific functions (Th1, Th2, Th17, and T-reg) (87). The normal role of T-reg cells is to balance the immune response. However, in most cancers, T-reg lymphocytes facilitate the immune escape of tumor cells, worsening prognosis and treatment response. On the contrary, in GI tract tumors, particularly CRC and EAC, the presence of T-reg cells has been associated with a favorable prognosis (88). The prevailing hypothesis is that T-reg cells exert an inhibitory effect on Th17 lymphocytes, historically characterized by pro-tumorigenic activity (89). Th1 and Th2 cells also play a fundamental role in the neoplastic progression from BE to EAC. Th1 cells, characterized by a pro-inflammatory profile, produce TNF-alpha and IFN-gamma, which induce cell-mediated immunity (e.g., natural killer [NK] cells), and then mediate anti-tumor activity (90). In contrast, Th2 cells release immunosuppressive cytokines (e.g., IL-4, IL-6, and IL-10), enhancing tumor progression. During EAC carcinogenesis, the Th1–Th2 cell homeostasis is altered. After an initial pro-inflammatory drive during esophagitis, sustained by IL-1β and IL-8, there is a shift to a Th2-like response with increased levels of IL-10 and IL-4. This shift suppresses the cell-mediated anti-tumor response (91). Other important components of the TME are macrophages and neutrophils, which can polarize into two different forms: an anti-tumor and a tumor-supporting phenotype (92). Recent evidence has highlighted the importance of not only identifying the immune infiltration phenotype but also understanding its location within the tumor core. Several studies have shown that higher T-cell infiltration in the tumor core is linked to better chemotherapy response and improved prognosis in contrast to localization at the peripheral margins of the tumor (93). Understanding and profiling the TME and its components are fundamental for the development of personalized therapy.

#### 3.4.1 TME and neoadjuvant chemotherapy

Standard chemotherapy (CT) and radiotherapy (RT) for EAC can have different effects on the TME, which could implicate a substantial clinical impact (94). In 2022, Croft et al. published a prospective study using single-cell analysis that included EAC patients, both treatment-naive and those with neoadjuvant chemotherapy (NAC) (95). The TME of the NAC group showed a significant increase in B-cells, endothelial cells, and CAFs, along with a considerable reduction in NK and T cells, compared to treatment-naive patients (95). TME modification after NAC can also impact prognosis. In a recently published study, Koemans et al. evaluated the prognostic significance of EAC-associated CD3+, CD4+, CD8+, FoxP3+, and PD-L1 expression in 123 patients who underwent NA-CRT and curative resection (96). The results showed that high CD8+ density is an independent marker of worse prognosis in poor responders to neoadjuvant treatments, suggesting that these patients may require adjuvant therapy (96).

#### 3.4.2 TME and immunotherapy

Immune checkpoints, including programmed cell death protein 1 (PD-1), cytotoxic T lymphocyte-associated protein 4 (CTLA-4), lymphocyte activation gene 3 (LAG-3), T-cell immunoglobulin and mucin domain 3 (TIM-3), and inducible T-cell co-stimulator (ICOS), represent surface proteins overexpressed during the differentiation of effector T cells in T-reg in carcinogenesis (97). Immune checkpoint inhibitors (ICI) are the primary agents used in immunotherapy for EAC, and anti-PD-1 or anti-PDL-1 monoclonal antibodies were used in the first trials in palliative settings (98). NAC and RT enhance the expression of immune checkpoints, thereby also favoring the use of ICI in adjuvant settings (99). The Gemstone-303, MATTERHORN, and DANTE trials showed significant improvements in overall survival, progression-free survival, and pathological response in patients treated with combination therapy (100–102). In the PERFECT trial, van der Ende T et al. evaluated the efficacy and safety of atezolizumab (a PD-L1 inhibitor) combined with standard CRT in the management of resectable EAC (103). The authors also conducted exploratory translational analyses to identify biomarkers of ICI therapy responses. After on-treatment biopsies, the authors distinguished two groups of non-responders based on cytotoxic T-cell (CTL) infiltration of the TME and the expression of T-cell exhaustion genes (PDCD1, HAVCR2, LAG3, TIM-3, and LAG-3). Non-responder patients with higher CTL rates and expression of these genes might benefit from additional ICIs, such as TIM-3 and LAG-3 antibodies (103). Regarding the role of the TME in predicting the response to ICIs, the results of an Italian study showed that a complete response to PD-1 blockade was associated with increased antitumor tissue-resident memory CD39+ CD103+ CD8+ T cells and reduced T-reg and M2-like macrophages (104).

### 3.5 Proteomics

In the research of BE progression risk biomarkers, protein expression analysis also plays a crucial role. It has been shown that implementing histopathological assessment with p53 immunohistochemistry (IHC) may improve the diagnostic capacity in detecting dysplastic cells in BE samples and could be performed routinely (105). IHC p53 abnormalities were useful not only in distinguishing dysplastic from non-dysplastic BE but also in identifying an increased progression risk to EAC (106). A limitation of this tool is the lack of standardization in p53 IHC staining evaluation, unlike the standardized approach for HER2 (107). In this context, growing interest in p53 IHC as a surrogate for genomic mutations in TP53, as a risk stratification biomarker, has been shown (108). Redston et al. evaluated the role of p53 IHC as a BE progression factor in a retrospective cohort of 561 BE patients with or without known progression and then validated the results in a prospective cohort of 1,487 BE patients (109). Abnormal p53 IHC highly correlated with TP53 mutation status (90.6% agreement) and was strongly associated with neoplastic progression in both the retrospective and prospective cohorts, regardless of histologic diagnosis (*p* <.001) (109). The latest innovation in this field is the Tissue Cypher Barrett’s Esophagus Assay, a risk prediction assay already validated in multicenter settings (110). This assay performs multiplexed fluorescence imaging analysis to extract quantitative data on various tissue biomarkers, enabling multivariable classification into three different risk classes for progression to HGD/EAC. In a retrospective cohort study, Frei et al. enrolled 155 BE patients. Histopathological samples with p53 IHC were independently reviewed by three expert pathologists and tested by using the Tissue Cypher Assay (111). The risk prediction assay provided significant risk stratification in BE patients with LGD and identified around 55% of BE progressors that the experts had down-staged to non-dysplastic BE (111).

### 3.6 Metagenomics and meta-transcriptomics in BE and EAC development

In recent years, esophageal microbiota (EM), defined as the community of microorganisms that inhabit the esophagus, has gained growing interest for its potential role in the pathogenesis and prognosis of esophageal diseases (112). For this reason, there has been a significant development of advanced molecular and computational techniques to profile EM, such as 16S rRNA gene sequencing and internal transcribed spacer (ITS) region sequencing (113). The introduction of shotgun metagenomics (the sequencing of all DNAs in a sample to identify and analyze the complete microbial community) and meta-transcriptomics (the analysis of whole transcripts over the human organism) was driven by the need to overcome the limitations of the aforementioned techniques, such as the small number of analyzed genes and the difficulty in associating the transcription of a particular gene with the corresponding species (114).

#### 3.6.1 Barrett’s esophagus

Several pieces of evidence have shown that the EM of reflux esophagitis (RE) patients presents a clear shift from *Bacteroidetes* spp. to Gram-negative (G-) bacteria, such as *Fusobacteria*, *Proteobacteria*, and *Campylobacter* spp. (115). These bacteria are involved in the inflammatory cascade through the interaction between lipopolysaccharide (LPS) and toll-like receptor 4 (TLR-4), leading to pro-inflammatory cytokine activation (IL-8, IL-6, IL-1, TNF-alpha, and NOS). It has been shown that the same proinflammatory cytokines are involved in the metaplastic transition and progression to dysplasia. For this reason, the transition from G+ to G- bacteria could be one among the pivotal mechanisms underlying the metaplastic transition (116). These findings were confirmed by two well-designed studies. In the first study, Snider et al. described two main microbiome profiles of GERD patients (with or without BE), showing that the microbiome, associated with RE and BE, was enriched with G- and microaerophilic bacteria (*Fusobacteria* and *Proteobacteria*) (117). Subsequently, Lopetuso et al. confirmed these findings, showing a significant reduction of *Bacteroidetes* (*Prevotella* spp.) in metaplastic BE tissue compared to healthy tissue (118). In BE patients, the colonic microbiome also has a role, with an increased *Firmicutes*/*Bacteroidetes* ratio, which is involved in decreased lower esophageal sphincter (LES) tone and gastric motility (119).

#### 3.6.2 Esophageal adenocarcinoma

In recent years, research on modifications of the EM in EAC has focused on five different pathogenetic mechanisms: increased lactate production (linked to angiogenesis, immunotolerance, and metastasis), LES relaxation due to iNOS release, delayed gastric emptying via COX-2 expression, and a pro-inflammatory environment driven by NLRP3 inflammasome and TLR activation (120). In this context, Elliot et al. showed that EAC samples had an abundance of lactic-acid-producing bacteria, such as *Lactobacillus*, *Staphylococcus*, *Bifidobacterium*, and *Streptococcus* (121). Regarding the fungal microbiota, EAC samples exhibited a prevalence of *Candida albicans* and *Candida glabrata* (122). Results from a recent meta-analysis suggest that *Helicobacter pylori* may have a protective role against EAC, but further evidence is needed (123).

## 4 Discussion and future perspectives

BE represents a fearful complication of GERD and EAC is one of the leading oncologic death causes. Therefore, the need to explore the oncologic pathogenesis and to improve early diagnosis is fundamental. The advent of omics technologies has powered up our understanding of BE/EAC-related processes. The availability of a large amount of genetic data with almost limitless computational power, thanks to genome and transcriptome-wide analyses, has unveiled several risk loci in the human genome potentially correlated with BE development and progression. The recent advancements have shown how, beyond SNP identification and association with BE/EAC, genome-wide aberrations, like CNAs, deletions, and TOH, play a relevant role in carcinogenesis cascade. Epigenetic modifications as well, such as the global hypo-methylation profile in progression from BE to EAC, are very promising in the field of biomarker discovery. The development of useful and easy-to-assess biomarkers is pivotal in the research field of BE. The ability to discriminate BE patients highly suspected to progress to cancer compared to the low-risk population is crucial. Unfortunately, data are still unable to clearly identify a common pathway; some BE cases are seemingly slow to progress while others show a rapidly evolving attitude. cf-DNA and circulating miRNAs could represent two promising actors in BE biomarker assay development. One of the key aspects of BE/EAC risk assessment and biomarker identification is the sampling technique. The standard sampling method, with endoscopic biopsy and subsequent FFPE inclusion with IHC staining and assessment, is burdened by the low quantity of materials and wide inter-observer variability. Many devices have been developed, evaluating protein expression (Tissue Cypher) or epigenetic alterations (Cytosponge or Esochacap), in order to maximize cell retention, minimizing risk and costs. Another key point is the availability of sampling and analysis essays which could preserve the spatial distribution and morphology of the pre-neoplastic/neoplastic tissue. Tissue Cypher is a groundbreaking invention which allows to preserve the spatial distribution of the sample analyzed. Furthermore, spatial genomics and transcriptomics could have a huge role in the next future in overcoming these issues, improving the evaluation of spatial and temporal distribution of biomarkers in BE/EAC. All of the abovementioned essays are based on the implementation of several factors (genetic, epigenetic, protein expression, and environmental) contributing altogether to build risk models for every BE patient with the highest accuracy possible. The “multi-omics” approach is *de facto* by far more comprehensive than single-omics analysis. Several promising results have been reported in the field of metagenomics and meta-transcriptomics, highlighting how the EM could have a huge impact in esophageal carcinogenesis, particularly through the immune cross-regulation of transcriptional factors and metabolic products. New studies using advanced technologies for the study of EM and its interaction with TME in EB and EAC are eagerly awaited.

## ENDEAVOR consortium members

We acknowledge all the ENDEAVOR consortium members: Antwerp University, University Hospital Antwerp, Ospedale San Raffaele SRL, Trinity College Dublin & St. James’s Hospital, Karolinska Institutet & Karolinska University Hospital (KUH, Region Stockholm), Heinrich-Heine-Universitaet Düsseldorf, Universal Diagnostics Sociedad Anonima, Centre Hospitalier Regional et Universitaire de Lille (CHU), Universitaet Leipzig & Universitatsklinikum Leipzig, Stichting Free University Medical Center VUMC, Stichting Radboud Universitair Medisch Centrum, Region Hovedstaden, ZAS – Ziekenhuis aan de Stroom, AZ Delta Roeselare, UZ Ghent.

## Glossary

BE: Barrett’s esophagus
GERD: gastro-esophageal reflux disease
EAC: esophageal adenocarcinoma
IM: intestinal metaplasia
LES: lower esophageal sphincter
PPI: proton pump inhibitor
Hp.: *Helicobacter pylori*
WL: white light
NBI: narrow band imaging
LGD: low-grade dysplasia
HGD: high grade
PET-CT: positron emission tomography–computed tomography
EUS: endoscopic ultrasound
PGE2: prostaglandin-E2
NF-κB: nuclear factor-κB
scRNA-seq: single-cell RNA sequencing
HV: healthy volunteers
SCJ: squamo-columnar junction
GWAS: genome-wide association studies
SNPs: single-nucleotide polymorphisms
WGS: whole-genome sequencing
OR: odds ratio
MHC: major histocompatibility complex
COX: cyclooxygenase
FFPE: formalin-fixed paraffin-embedded biopsies
CNAs: copy-number alterations
AUC: area under the curve
TOH: tracts of homozygosity
IRs: inversed repeats
NGS: next-generation sequencing
miRNAs: micro-RNAs
GO: Gene Ontology
GI: gastrointestinal
TME: tumor microenvironment
ECM: extracellular matrix
NK: natural killer
CT: standard chemotherapy
RT: radiotherapy
NAC: neoadjuvant chemotherapy
PD-1: programmed cell death protein 1
CTLA-4: cytotoxic T lymphocyte-associated protein 4
LAG-3: lymphocyte-activation gene 3
TIM-3: T-cell immunoglobulin and mucin domain 3
ICOS: inducible T-cell co-stimulator
CTLs: cytotoxic T cells
IHC: immunohistochemistry
EM: esophageal microbiota
TLR-4: toll-like receptor 4
ROS: reactive oxygen species
PARP: poly(ADP-ribose) polymerase
PCR: polymerase chain reaction
